# Inhibited growth of colon cancer carcinomatosis by antibodies to vascular endothelial and epidermal growth factor receptors

**DOI:** 10.1054/bjoc.2001.1936

**Published:** 2001-08

**Authors:** R M Shaheen, S A Ahmad, W Liu, N Reinmuth, Y D Jung, W W Tseng, K E Drazan, C D Bucana, D J Hicklin, L M Ellis

**Affiliations:** Departments of Surgical Oncology and; Cancer Biology, The University of Texas MD Anderson Cancer Center, 1515 Holcombe Boulevard-444, Houston, Texas, 77030, USA; Department of Surgery, Stanford University, 1201 Welch Road, Palo Alto, California, 94304, USA; ImClone Systems Inc., 180 Varick Street, New York, New York, 10014, USA

**Keywords:** antiangiogenic therapy, VEGF, EGF, receptor, apoptosis, endothelial cell, DC101, C225

## Abstract

Vascular endothelial growth factor (VEGF) and epidermal growth factor (EGF) regulate colon cancer growth and metastasis. Previous studies utilizing antibodies against the VEGF receptor (DC101) or EGF receptor (C225) have demonstrated independently that these agents can inhibit tumour growth and induce apoptosis in colon cancer in in vivo and in vitro systems. We hypothesized that simultaneous blockade of the VEGF and EGF receptors would enhance the therapy of colon cancer in a mouse model of peritoneal carcinomatosis. Nude mice were given intraperitoneal injection of KM12L4 human colon cancer cells to generate peritoneal metastases. Mice were then randomized into one of four treatment groups: control, anti-VEGFR (DC101), anti-EGFR (C225), or DC101 and C225. Relative to the control group, treatment with DC101 or with DC101+C225 decreased tumour vascularity, growth, proliferation, formation of ascites and increased apoptosis of both tumour cells and endothelial cells. Although C225 therapy did not change any of the above parameters, C225 combined with DC101 led to a significant decrease in tumour vascularity and increases in tumour cell and endothelial cell apoptosis (vs the DC101 group). These findings suggest that DC101 inhibits angiogenesis, endothelial cell survival, and VEGF-mediated ascites formation in a murine model of colon cancer carcinomatosis. The addition of C225 to DC101 appears to lead to a further decrease in angiogenesis and ascites formation. Combination anti-VEGF and anti-EGFR therapy may represent a novel therapeutic strategy for the management of colon peritoneal carcinomatosis. © 2001 Cancer Research Campaign http://www.bjcancer.com


*British Journal of Cancer* (2001) **85**(4), 584–589
© 2001 Cancer Research Campaign
doi: 10.1054/ bjoc.2001.1936, available online at http://www.idealibrary.com on

### Inhibited growth of colon cancer carcinomatosis by antibodies to vascular endothelial and epidermal growth factor receptors

**RM Shaheen[1], SA Ahmad[1], W Liu[2], N Reinmuth[2], YD Jung[2], WW Tseng[3], KE Drazan[3], CD Bucana[2], DJ Hicklin[4] and LM Ellis[1,2]**

Departments of [1]Surgical Oncology and [2]Cancer Biology, The University of Texas MD Anderson Cancer Center, 1515 Holcombe Boulevard-444, Houston, Texas, 77030, USA; [3]Department of Surgery, Stanford University, 1201 Welch Road, Palo Alto, California, 94304, USA; and [4]ImClone Systems, Inc., 180 Varick Street, New York, New York, 10014, USA

**Summary** Vascular endothelial growth factor (VEGF) and epidermal growth factor (EGF) regulate colon cancer growth and metastasis. Previous studies utilizing antibodies against the VEGF receptor (DC101) or EGF receptor (C225) have demonstrated independently that these agents can inhibit tumour growth and induce apoptosis in colon cancer in in vivo and in vitro systems. We hypothesized that simultaneous blockade of the VEGF and EGF receptors would enhance the therapy of colon cancer in a mouse model of peritoneal carcinomatosis. Nude mice were given intraperitoneal injection of KM12L4 human colon cancer cells to generate peritoneal metastases. Mice were then randomized into one of four treatment groups: control, anti-VEGFR (DC101), anti-EGFR (C225), or DC101 and C225. Relative to the control group, treatment with DC101 or with DC101+C225 decreased tumour vascularity, growth, proliferation, formation of ascites and increased apoptosis of both tumour cells and endothelial cells. Although C225 therapy did not change any of the above parameters, C225 combined with DC101 led to a significant decrease in tumour vascularity and increases in tumour cell and endothelial cell apoptosis (vs the DC101 group). These findings suggest that DC101 inhibits angiogenesis, endothelial cell survival, and VEGF-mediated ascites formation in a murine model of colon cancer carcinomatosis. The addition of C225 to DC101 appears to lead to a further decrease in angiogenesis and ascites formation. Combination anti-VEGF and anti-EGFR therapy may represent a novel therapeutic strategy for the management of colon peritoneal carcinomatosis. © 2001 Cancer Research Campaign http://www.bjcancer.com

**Keywords**: antiangiogenic therapy; VEGF; EGF; receptor; apoptosis; endothelial cell; DC101; C225

Tumour growth and dissemination are angiogenesis-dependent processes (Folkman, 1995). One anti-angiogenic strategy targets known mediators of angiogenesis in specific tumours. In colon cancer, the over-expression of vascular endothelial growth factor (VEGF) and its tyrosine kinase receptor correlates with the development of metastases (Takahashi et al, 1995). DC101, a monoclonal neutralizing antibody that targets the mouse VEGF receptor-2 (flk-1), blocks ligand binding and receptor signaling in vitro, inhibits the growth and vascularity of tumours in murine models of colon cancer liver metastasis, and can induce apoptosis of endothelial cells (ECs) and tumour cells (TCs) in vivo (Bruns et al, 2000a).

Epidermal growth factor (EGF) is a known regulator of colon cancer proliferation in vitro, and over-expression of the EGF receptor correlates with the development of colon cancer metastases (Ciardiello et al, 1999; Radinsky et al, 1995). A recent study involving use of a humanized chimeric mouse monoclonal antibody directed against the EGF receptor (C225) showed that inhibiting the activity of EGF can induce TC apoptosis in human colon cancer cells (Liu et al, 2000).

In addition to liver and regional lymph nodes, colon cancer also metastasizes to the peritoneal cavity; peritoneal carcinomatosis, the resulting condition, occurs in 12–20% of patients whose disease recurs (Copeland et al, 1968; Marcus et al, 1999). Peritoneal carcinomatosis is associated with significant morbidity and mortality from the formation of ascites and bowel obstruction. Given the significant antitumour effect of C225 against colon cancer cells in vitro and that of DC101 in previous studies of colon cancer metastases in vivo, we hypothesized that combination therapy with DC101, which inhibits the VEGF receptor and affects endothelial cells, and C225, which inhibits the EGF receptor and affects tumour cells, would be a novel, efficacious therapeutic strategy against peritoneal carcinomatosis because it targets several cell types within the tumour microenvironment.

The primary purpose of this study was to determine the effects of selective blockade of the VEGF and EGF receptors on tumour angiogenesis, growth, and ascites formation in a murine model of human colon cancer peritoneal carcinomatosis. A secondary goal was to assess whether combination therapy with DC101 and C225 would demonstrate additive or synergistic effects on these variables.

*Received 2 October 2000*
*Revised 24 April 2001*
*Accepted 26 April 2001*

*Correspondence to: LM Ellis*

### MATERIALS AND METHODS

### Reagents and antibodies

Reagents were obtained as follows: minimal essential medium (MEM), fetal bovine serum (FBS), penicillin-streptomycin, vitamins, sodium pyruvate, L-glutamine, nonessential amino acids, trypsin-EDTA, Hanks balanced salt solution (HBSS), and Trypan blue from Life Technologies, Inc. (Grand Island, NY); 27- and 30-gauge needles, 1-ml syringes, and Gill 3 haematoxylin from Sigma Chemical Company (St. Louis, MO); optimum cutting temperature (OCT) compound from Miles Inc. (Elkhart, IN); diaminobenzidine (DAB) substrate and Universal Mount from Research Genetics (Huntsville, AL); Superfrost slides from Fisher Scientific Co. (Houston, TX); TUNEL kit from Promega (Madison, WI); and 4,6-diamidino-2-phenylindole dihydrochloride (DAPI) mount from Vector Laboratories, Inc. (Burlingame, CA).

Antibodies for immunohistochemical analysis and antiangiogenic therapy were obtained as follows: rat anti-mouse CD31/PECAM-1 antibody from Pharmingen (San Diego, CA); mouse anti-proliferating cell nuclear antigen (PCNA) clone PC10 DAKO A/S from DAKO Corp. (Carpinteria, CA); peroxidase-conjugated goat anti-rat immunoglobulin (IgG) (H+L) and Texas Red-conjugated goat anti-rat IgG from Jackson Research Laboratories (West Grove, PA); peroxidase-conjugated rat anti-mouse IgG2a from Serotec Harlan Bioproducts for Science, Inc. (Indianapolis, IN); and rat anti-mouse VEGF receptor-2 monoclonal antibody (Prewett et al, 1999; Witte et al, 1998) and chimeric anti-human EGF receptor monoclonal antibody (Goldstein et al, 1995) from ImClone Systems, Inc (New York, NY) (Prewett et al, 1999; Witte et al, 1998).

### Cell culture

The human colon cancer cell line KM12L4 is a metastatic variant of KM12C (established from a Dukes stage B2 surgical specimen of colon cancer) selected by repeated intrasplenic injections in nude mice (Morikawa et al, 1988). This cell line was the generous gift of Dr Isaiah J. Fidler (The University of Texas M D Anderson Cancer Center, Houston, TX). Cells were cultured and maintained in MEM supplemented with 10% FBS and harvested from subconfluent cultures as described elsewhere (Bruns et al, 2000a).

### Animals and tumour cell inoculation

Eight-week-old male athymic nude mice (National Cancer Institute, Animal Production Area, Frederick, MD), caged in groups of five, were acclimatized for 1 week. Then, each mouse was given an intraperitoneal injection of $1.0 \times 10^6$ KM12L4 colon cancer cells in 500 µl of HBSS with a 30-gauge needle attached to a 1-ml syringe. Mice were then randomized into one of four treatment groups (10 mice per group): control, DC101, C225, or DC101 and C225. All animal studies were conducted under guidelines approved by the Animal Care and Use Committee of MD Anderson Cancer Center and UKCCCR guidelines (UKCCCR, 1998).

### Administration of antibodies

Beginning 3 days after injection of tumour cells, mice were given intraperitoneal (i.p.) injections every third day of either control vehicle (phosphate buffered saline [PBS]), DC101 (0.8 mg), C225 (1.0 mg), or DC101 (0.8 mg) and C225 (1.0 mg) (each injection given in a 700-µl total volume) with a 27-gauge needle attached to a 1-ml syringe. Mice were weighed weekly. Mice were killed when the control group became moribund (about 30 days after therapy began), and complete necropsies were performed, tumour burden was quantified, and tumours were harvested and analysed as described below.

### Necropsy and tissue preparation

Mice were killed by $CO_2$ asphyxiation and weighed. For each mouse, necropsy was performed, the size of peritoneal tumours was measured with calipers, the mean tumour size was determined, and representative lesions were excised. The extent of ascites was assessed by an investigator who was unaware of the treatment-group assignment as follows: grade 0, no ascites; grade 1, small ascites; grade 2, moderate ascites; grade 3, large ascites; grade 4, massive ascites with tense abdomen (Aparicio et al, 1999). Tumour sections were either embedded in OCT compound and frozen at –70˚C or fixed in formalin and then embedded in paraffin.

### Immunohistochemical analyses of paraffin-embedded and frozen tissues

Tissue sections were treated by standard deparaffinization (for formalin-fixed and paraffin-embedded tissues) or by fixation in acetone and chloroform (for tissues frozen in OCT), and immunohistochemical analyses were performed as described previously (Shaheen et al, 1999). Briefly, endogenous peroxidases were blocked with 3% $H_2O_2$ in methanol, and the slides were washed with PBS, incubated for 20 min in protein-blocking solution (PBS supplemented with 1% normal goat serum and 5% normal horse serum), and incubated overnight at 4˚C with primary antibodies against CD31 or PCNA. Then, the slides were washed, incubated with protein-blocking solution, incubated for 1 h at room temperature with peroxidase-conjugated secondary antibodies, washed, incubated with DAB, washed, counterstained with haematoxylin, washed, and mounted with Universal Mount and dried on a hot plate at 56˚C. Frozen sections to be stained for CD31 were incubated with a secondary antibody conjugated to Texas Red (red fluorescence) instead of the peroxidase-conjugated antibody. Omission of the primary antibody served as negative control.

### TUNEL assay for apoptotic cells

Terminal deoxynucleotidyl transferase-(TdT)-mediated dUTP nick-end labelling (TUNEL) staining was performed according to the manufacturer's protocol. Briefly, the sections were fixed with 4% methanol-free paraformaldehyde, washed, permeabilized with 0.2% Triton X-100, washed, incubated with the kit's equilibration buffer, incubated with a reaction mix containing equilibration buffer, nucleotide mix, and the TdT enzyme at 37˚C for 1 h, incubated for 15 min at room temperature with 2 × standard saline citrate to stop the TdT reaction, washed, stained with DAPI mount (to visualize the nuclei), and glass coverslips were applied.

### Quantification of CD31 (endothelial cells or tumour vessels), PCNA (tumour cell proliferation), and apoptotic cells

Numbers of tumour vessels and PCNA-positive cells were evaluated by light microscopy (counted in five random 0.159- $mm^2$ fields at 100× magnification), imaged digitally, and processed with Optimas Image Analysis software (Biscan, Edmond, WA). Apoptotic cells were visualized with immunofluorescence as follows. Sections were digitally imaged and processed with Adobe Photoshop software (Adobe Systems, Mountain View, CA). CD31-positive ECs were detected by localized red fluorescence by

© 2001 Cancer Research Campaign using a rhodamine filter. Apoptosis was determined by localized green fluorescence (for TCs) or green with red fluorescence (for ECs) by using a fluorescein filter. Nuclei were detected by the blue fluorescence of the DAPI with its filter. Cells were counted in five consecutive, non- overlapping fields 0.011-$mm^2$ fields per slide at 400× magnification with the first field selected at random in a non-necrotic portion of the tumour. The percentages of apoptotic ECs and TCs per field were then calculated as [% apoptotic cells = (number of apoptotic cells / total number of cells) × 100].

### Statistical analysis

All comparisons were tested for statistical significance with the Mann-Whitney U-test (InStat Statistical Software, San Diego, CA). $P \leq 0.05$ was considered statistically significant.

### RESULTS

### Gross tumour burden and ascites formation

Body weight at the termination of the experiment was similar in the treatment and control groups, and no obvious toxic effects attributable to DC101 or C225 therapy were noted. Necropsy confirmed the presence of peritoneal metastases in 100% of the control mice.

Gross tumour burden was assessed by measuring the diameter of peritoneal lesions 30 days after therapy was begun (Figure 1A). Mean peritoneal tumour size was smaller in the DC101 group (50.3%, $P = 0.006$) and in the combination DC101 + C225 group (66.7%, $P = 0.001$) than in the control group. No differences were found between the control and C225 groups ($P = 0.842$) or between the DC101 and DC101 + C225 groups ($P = 0.248$). Although 100% of control and C225 mice had peritoneal disease at the end of the study, 10% of DC101 mice (1 of 10 mice) and 30% of the combination-therapy mice (3 of 10 mice) showed no evidence of disease (not significantly different by chi-square analysis).

Ascites was graded on a 0 to 4 scale (Figure 1B). Mean ascites grade was lower for both the DC101 (66.7%, $P < 0.001$), and combination-therapy (100%, $P < 0.001$) groups than for the control mice. No difference was found between the control and C225 groups ($P = 0.842$). Moreover, the DC101 + C225 group had significantly less ascites than did the DC101 group ($P < 0.001$); virtually no ascites was found in the combination-therapy group.

### Tumour cell proliferation

Tissue sections were also stained for PCNA by immunohistochemical analysis to assess tumour cell proliferation (Figure 2). Peritoneal tumours were smaller in the DC101 (50.3%, $P = 0.006$) and DC101+C225 groups (66.7%, $P = 0.001$) than in the control group. No differences were found between the control and C225 groups ($P = 0.842$) or between the DC101 and DC101 + C225 groups ($P = 0.248$).

### Angiogenesis

Sections of peritoneal lesions from mice in all four treatment groups were stained for CD31 immunofluorescence to detect the number of ECs as a measure of tumour angiogenesis (Figure 3). Significantly fewer ECs was observed in the DC101 ($P < 0.001$)

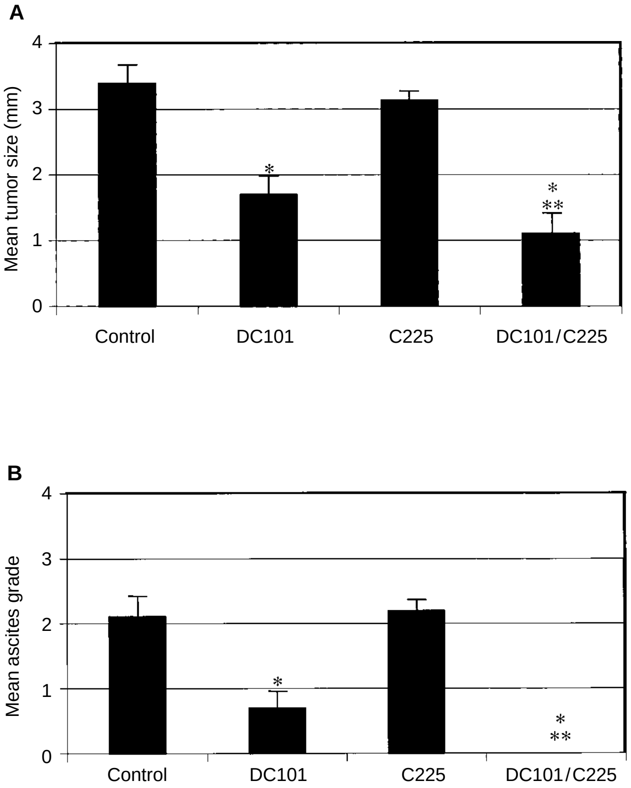

**Figure 1** Effect of DC101 and C225 on gross tumour burden and ascites formation in mice with colon cancer peritoneal carcinomatosis. (**A**) Peritoneal tumours in the DC101 and DC101 + C225 treatment groups were significantly smaller than those in the control mice (bars, SE; *$P < 0.006$ vs control, **$P < 0.001$ vs control). C225 had no demonstrable anti-tumour effects when given alone or in combination with DC101. (**B**) Significantly less ascites formed in the DC101 and DC101 + C225 treatment groups relative to that in the control mice (bars, SE; *$P < 0.001$ vs control; **$P < 0.001$ vs DC101). Although C225 did not inhibit ascites when given alone, C225 augmented the effect of DC101

and DC101+ C225 groups ($P < 0.001$) than in the control group. Although no difference was found between the control and C225 groups ($P = 0.436$), fewer ECs were present in the DC101+ C225 group (41.2%, $P < 0.023$) than in the DC101 group.

### Apoptosis of tumour cells and endothelial cells

Immunofluorescent TUNEL staining, with and without concurrent staining for CD31, was performed on sections of peritoneal metastases to quantify TC (Figure 4A) and EC (Figure 4B) apoptosis. More apoptotic TCs were observed in the DC101 (14.3-fold, $P < 0.001$) and DC101+ C225 groups (23.6-fold, $P < 0.001$) than in the control group. Although no difference was found between the control and C225 groups ($P = 0.796$), more apoptotic TCs were present in the DC101+C225 group (1.7-fold, $P = 0.004$) than in the DC101 group. Similar results were found for the number of apoptotic ECs: More apoptotic ECs were present in the DC101 (226-fold, $P < 0.001$) and DC101 + C225 groups (331-fold, $P < 0.001$) than in the control group. Although no difference was found between the control and C225 groups ($P = 0.739$), more apoptotic ECs were found in the DC101 + C225 group (1.46-fold, $P < 0.043$) than in the DC101 group.

Immunoflurescent images of tumour sections, illustrating the relative amounts of TC and EC apoptosis, are shown in Figure 4C.

*© 2001 Cancer Research Campaign*

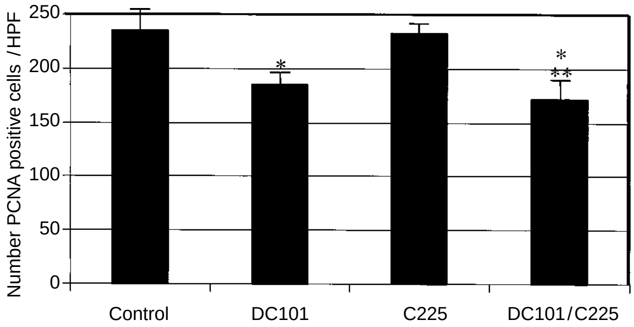

**Figure 2** Effect of DC101 and C225 on tumour cell proliferation. Immunohistochemical staining of peritoneal tumour sections for PCNA (a measure of tumour cell proliferation) showed that DC101 and DC101 + C225 therapy significantly inhibited tumour cell proliferation (*$P < 0.001$ vs control). C225, given alone, did not affect tumour cell proliferation

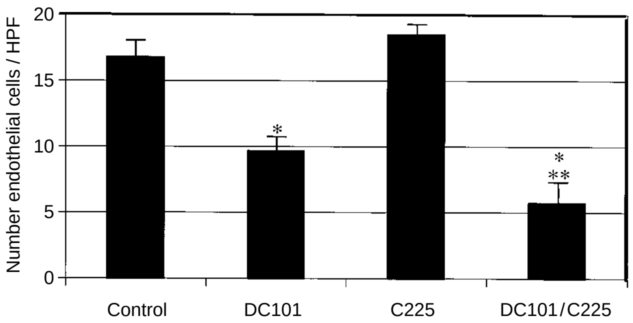

**Figure 3** Effect of DC101 on tumour angiogenesis. Immunofluorescent staining of peritoneal tumour sections for CD31 demonstrated that DC101 and DC101+C225 significantly inhibited tumour vascularity (*$P < 0.001$ vs control). C225 did not demonstrate significant antiangiogenic effects when administered alone but did significantly augment the effect of DC101 (**$P = 0.023$ vs DC101 alone). Bars, SEM

### DISCUSSION

Approximately 180 000 new cases of colon cancer are diagnosed each year in the United States alone, and about half of these patients eventually develop metastases. Given the prevalence of colon cancer and the high morbidity and mortality associated with peritoneal carcinomatosis, finding effective treatments for these conditions is essential. Recent advances in understanding the biology of colon cancer metastases have shown that VEGF and EGF regulate tumour growth, proliferation, and disease progression (Ciardiello et al, 1999; Liu et al, 2000; Takahashi et al, 1995) and have made it possible to devise novel biologically based therapies that may inhibit the activity of mediators of this disease.

In this report, we used a murine model of human colon cancer peritoneal carcinomatosis to determine whether selective blockade of the VEGF receptor-2 (by DC101) or the EGF receptor (by C225) would alter tumour growth kinetics and affect EC and TC survival. Treatment with DC101 decreased tumour growth, tumour cell proliferation and vascularity and significantly increased EC and TC apoptosis. These findings were consistent with those from another study done in our laboratory with a CT-26 colon cancer cell line syngeneic to BALB/c mice (unpublished data, 2000). In that study, we showed that VEGF is an in vivo EC survival factor for peritoneal lesions. In temporal studies, we found that inhibiting VEGF activity leads to an initial induction of EC apoptosis and, with continuing therapy, a subsequent induction of TC apoptosis.

Other recent findings revealed that TC apoptosis of human colon cancer cells was induced by C225 treatment in vitro (Liu et al, 2000). On the basis of these in vitro findings, we hypothesized that administration of C225 in our in vivo model of colon carcinomatosis would also induce TC apoptosis. However, we found no significant induction in TC or EC apoptosis from C225 therapy, a finding that contrasts with those of others showing that C225 led to a decrease in angiogenesis and an associated increase in TC apoptosis (Bruns et al, 2000b; Perrotte et al, 1999). The difference in these results probably relates to differences in the tumour model or system studied. However, in our study, C225 did potentiate the effects of DC101 by significantly increasing TC and EC apoptosis relative to that produced by DC101 alone. Our current findings indicate that an EGF receptor blockade alone is not sufficient for inducing TC and EC apoptosis in this model, but in the presence of a VEGF inhibitor (DC101), EGF receptor blockade can significantly augment DC 101's induction of TC and EC apoptosis.

In addition to potentiating the effect of DC101 on TC apoptosis, C225 also augmented DC101's induction of EC apoptosis and its inhibition of angiogenesis. Since C225 does not effect mouse EGF-R, it is likely that whatever effect C225 had on the tumours was indirect, mediated by alterations in the tumour cells themselves as stated above.

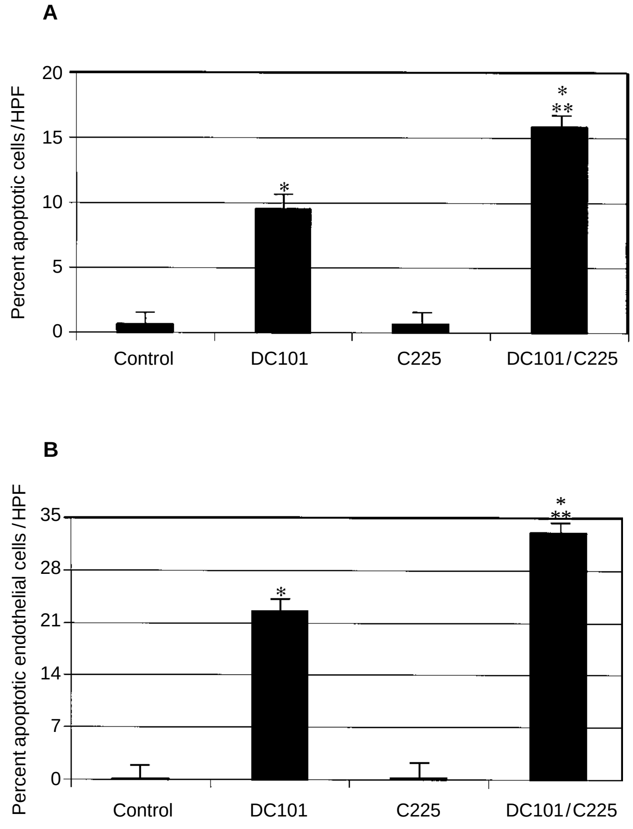

**Figure 4** Effect of DC101 on apoptosis of tumour cells and endothelial cells. Immunofluorescent staining for (**A**) TUNEL (TC apoptosis) and (**B**) sequential staining for CD31 plus TUNEL (EC apoptosis) were performed in peritoneal tumour sections and the mean numbers of positive-staining cells (expressed as a percentage of the total number of cells) were determined. DC101 and DC101 + C225 therapy significantly increased apoptosis of both TCs and ECs. Given alone, C225 did not induce apoptosis but significantly augmented DC101's induction of both TC and EC apoptosis (by 65.4% and 46.4%, respectively). (Bars, SEM; *$P < 0.001$ vs control; **$P < 0.043$ vs control)

*© 2001 Cancer Research Campaign*

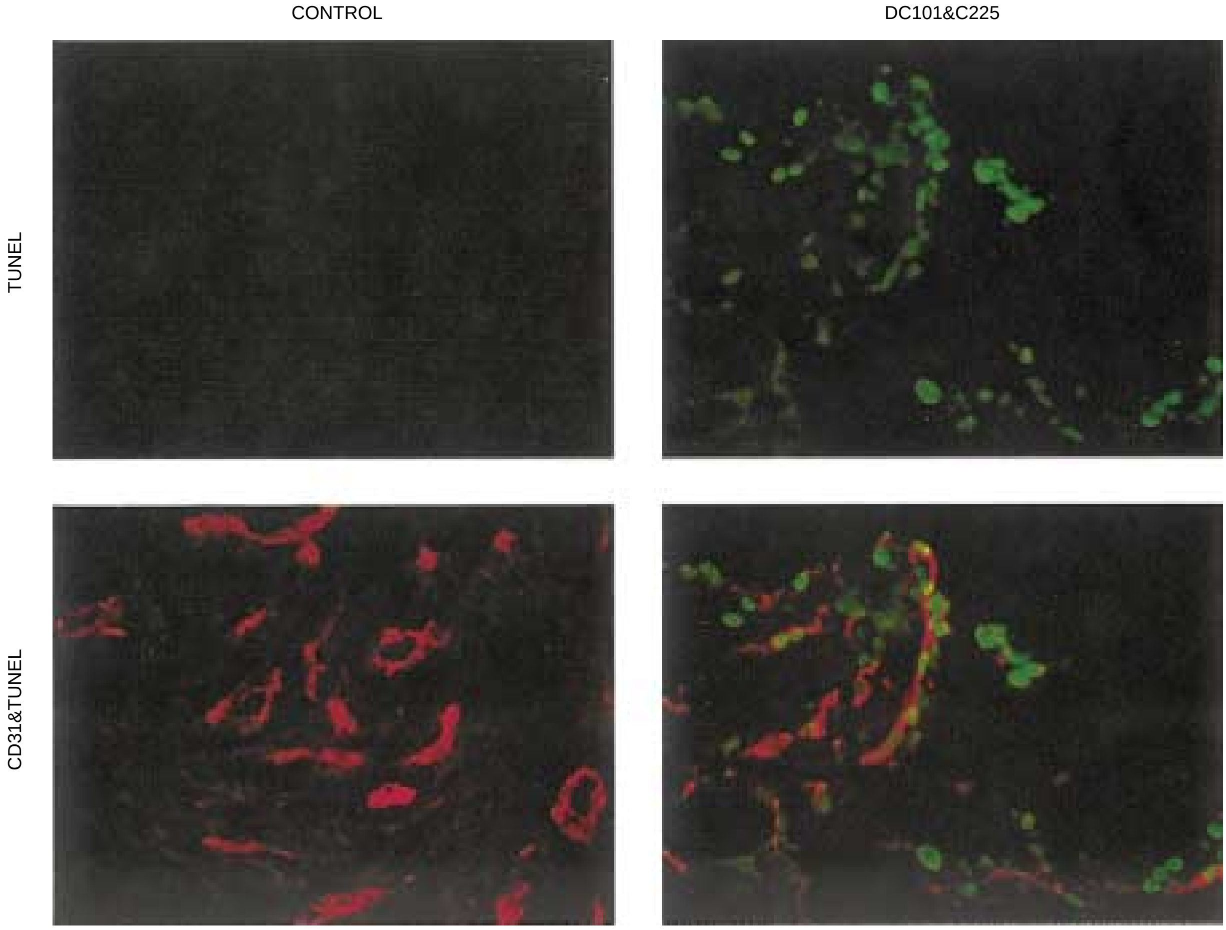

**Figure 5** Representative immunofluorescent images for TUNEL (top row, 200 × ) and for CD31 plus TUNEL (bottom row, 200×) after 30 days of treatment for the control group (treated with PBS) (column 1) and the DC101 + C225 group (column 2) show enhanced apoptosis of both TCs and ECs in the treatment group

Given the 100% tumorigenicity of this model in control mice, our finding that 10% of the DC101-treated mice and 30% of the DC101+C225-treated mice had no evidence of disease at the termination of the study suggests that these mice had a complete clinical response to therapy. However, this apparent decrease in tumorigenicity was not borne out in statistical analyses relative to controls. With regard to the formation of ascites, DC101 and the combination of DC101 and C225 greatly reduced the extent of ascites relative to that in the control group (by 66.7% and 100.0%, respectively). This finding indirectly affirms the potency of VEGF as a permeability factor (Senger et al, 1990). Others have shown that anti-VEGF therapy can inhibit vascular permeability induced by various malignancies (Ke et al, 1996; Zebrowski et al, 1999). The mechanism by which C225 enhanced the ability of DC101 to inhibit ascites formation is unknown.

In conclusion, we have shown that selective blockade of the VEGF receptor-2 by DC101 significantly decreased tumour vascularity, growth, proliferation, and ascites formation, and increased EC and TC apoptosis in a relevant model of human colon cancer peritoneal carcinomatosis. Moreover, tumour vascularity, ascites formation, and EC and TC apoptosis were successfully augmented by the concurrent blockade of the EGF receptor by C225. We conclude that the combined inhibition of VEGF and EGF activity, a novel biologically based anti-neoplastic therapeutic strategy, has broad anti-tumour effects that may have clinical utility in managing peritoneal carcinomatosis in humans.

### ACKNOWLEDGEMENTS

We thank Christine F. Wogan of the Department of Scientific Publications and Joan Small of the Department of Surgical Oncology for editorial assistance. Supported by NIH grant T32 CA 09599 [RMS, SAA], grants from the Gillson Longenbaugh Foundation and the Jon and Suzie Hall Fund for Colon Cancer Research [LME], and NIH Cancer Center Support Core Grant CA16672.

### REFERENCES

American Cancer Society (2000) Cancer facts and figures. http://www.cancer.org/statistics/cff2000/selectedcancers.html#col

Aparicio T, Kermorgant S, Dessirier V, Lewin MJ and Lehy T (1999) Matrix metalloproteinase inhibition prevents colon cancer peritoneal carcinomatosis development and prolongs survival in rats. *Carcinogenesis* **20**: 1445–1451

Bruns CJ, Liu W, Davis DW, Shaheen RM, McConkey DJ, Wilson MR, Bucana CD, Hicklin DJ and Ellis LM (2000a) Vascular endothelial growth factor is an in vivo survival factor for tumor endothelium in a murine model of colorectal carcinoma liver metastases. *Cancer* **89**: 488–499

Bruns CJ, Solorzano CC, Harbison MT, Ozawa S, Tsan R, Fan D, Abbruzzese J, Traxler P, Buchdunger E, Radinsky R and Fidler IJ (2000b) Blockade of the

 *© 2001 Cancer Research Campaign*

epidermal growth factor receptor signaling by a novel tyrosine kinase inhibitor leads to apoptosis of endothelial cells and therapy of human pancreatic carcinoma. *Cancer Res* **60**: 2926–2935

Ciardiello F, Bianco R, Damiano V, De Lorenzo S, Pepe S, De Placido S, Fan Z, Mendelsohn J, Bianco AR and Tortora G (1999) Antitumor activity of sequential treatment with topotecan and anti-epidermal growth factor receptor monoclonal antibody C225. *Clin Cancer Res* **5**: 909–916

Copeland EM, Miller LD and Jones RS (1968) Prognostic factors in carcinoma of the colon and rectum. *Am J Surg* **116**: 875–881

Folkman J (1995) Seminars in Medicine of the Beth Israel Hospital, Boston. Clinical applications of research on angiogenesis [see comments]. *N Engl J Med* **333**: 1757–1763

Goldstein NI, Prewett M, Zuklys K, Rockwell P and Mendelsohn J (1995) Biological efficacy of a chimeric antibody to the epidermal growth factor receptor in a human tumor xenograft model. *Clin Cancer Res* **1**: 1311–1318

Ke L, Qu H, Nagy JA, Eckelhoefer IA, Masse EM, Dvorak AM and Dvorak HF (1996) Vascular targeting of solid and ascites tumours with antibodies to vascular endothelial growth factor. *Eur J Cancer* **32A**: 2467–2473

Liu B, Fang M, Schmidt M, Lu Y, Mendelsohn J and Fan Z (2000) Induction of apoptosis and activation of the caspase cascade by anti-EGF receptor monoclonal antibodies in DiFi human colon cancer cells do not involve the c-jun N-terminal kinase activity. *Br J Cancer* **82**: 1991–1999

Marcus EA, Weber TK, Rodriguez-Bigas MA, Driscoll D, Meropol NJ and Petrelli NJ (1999) Prognostic factors affecting survival in patients with colorectal carcinomatosis. *Cancer Invest* **17**: 249–252

Morikawa K, Walker SM, Jessup JM and Fidler IJ (1988) In vivo selection of highly metastatic cells from surgical specimens of different primary human colon carcinomas implanted into nude mice. *Cancer Res* **48**: 1943–1948

Perrotte P, Matsumoto T, Inoue K, Kuniyasu H, Eve BY, Hicklin DJ, Radinsky R and Dinney CP (1999) Anti-epidermal growth factor receptor antibody C225 inhibits angiogenesis in human transitional cell carcinoma growing orthotopically in nude mice. *Clin Cancer Res* **5**: 257–265

Prewett M, Huber J, Li Y, Santiago A, O'Connor W, King K, Overholser J, Hooper A, Pytowski B, Witte L, Bohlen P and Hicklin DJ (1999) Antivascular endothelial growth factor receptor (fetal liver kinase 1) monoclonal antibody inhibits tumor angiogenesis and growth of several mouse and human tumors. *Cancer Res* **59**: 5209–5218

Radinsky R, Risin, Fan, Dong, Bielenberg, Bucana and Fidler (1995) Level and function of epidermal growth factor receptor predict the metastatic potential of human colon carcinoma cells. *Clin Cancer Res* **1**: 19–31

Senger DR, Connolly DT, Van de Water L, Feder J and Dvorak HF (1990) Purification and NH2-terminal amino acid sequence of guinea pig tumor-secreted vascular permeability factor. *Cancer Res* **50**: 1774–1778

Shaheen RM, Davis DW, Liu W, Zebrowski BK, Wilson MR, Bucana CD, McConkey DJ, McMahon G and Ellis LM (1999) Antiangiogenic therapy targeting the tyrosine kinase receptor for vascular endothelial growth factor receptor inhibits the growth of colon cancer liver metastasis and induces tumor and endothelial cell apoptosis. *Cancer Res* **59**: 5412–5416

Takahashi Y, Kitadai Y, Bucana CD, Cleary KR and Ellis LM (1995) Expression of vascular endothelial growth factor and its receptor, KDR, correlates with vascularity, metastasis, and proliferation of human colon cancer. *Cancer Res* **55**: 3964–3968

United Kingdom Co-ordinating Committee on Cancer Research (UKCCCR) (1998) Guidelines for the welfare of animals in experimental neoplasia (second edition) *Br J cancer 77: 1–10*

Witte L, Hicklin DJ, Zhu Z, Pytowski B, Kotanides H, Rockwell P and Bohlen P (1998) Monoclonal antibodies targeting the VEGF receptor-2 (Flk1/KDR) as an anti-angiogenic therapeutic strategy. *Cancer Metastasis Rev* **17**: 155–161

Zebrowski BK, Yano S, Liu W, Shaheen RM, Hicklin DJ, Putnam JB, Jr and Ellis LM (1999) Vascular endothelial growth factor levels and induction of permeability in malignant pleural effusions. *Clin Cancer Res* **5**: 3364–3368

*© 2001 Cancer Research Campaign*
